# Outcome of revascularization therapy in traumatized immature incisors

**DOI:** 10.1186/s12903-020-01193-5

**Published:** 2020-07-14

**Authors:** Carolina W. Mittmann, Eckehard Kostka, Husam Ballout, Mareike Preus, Robert Preissner, Murat Karaman, Saskia Preissner

**Affiliations:** 1 Department of Operative and Preventive Dentistry, Charité – Universitätsmedizin Berlin, Corporate member of Freie Universität Berlin, Humboldt-Universität zu Berlin, and Berlin Institute of Health, Assmannshauser Straße 4-6, 14197 Berlin, Germany; 2 Institute of Physiology, Charité – Universitätsmedizin Berlin, Corporate member of Freie Universität Berlin, Humboldt-Universität zu Berlin, and Berlin Institute of Health, Philippstrasse 12, 10115 Berlin, Germany; 3 Department Oral, Maxillary and Maxillofacial Surgery, Charité – Universitätsmedizin Berlin. Corporate member of Freie Universität Berlin, Humboldt-Universität zu Berlin, and Berlin Institute of Health, Augustenburger Platz 1, 13353 Berlin, Germany

**Keywords:** Dental trauma, Avulsion, intrusion, Luxation, Revascularization

## Abstract

**Background:**

The aim of this retrospective analysis was to evaluate the clinical and radiological outcome of revascularization therapy in traumatized permanent incisors to determine whether this approach could be implemented into clinical routine.

**Methods:**

A total of 16 traumatized incisors (either avulsion or severe luxation/intrusion) with open apices (> 1 mm) that underwent revascularization following a standardized protocol were analyzed with a mean follow-up of 22 months. Radiographs and clinical parameters (such as root length, pulp space, dentin wall width, apical foramen, alveolar bone loss, ankylosis/mobility, supra−/infraposition, discoloration, probing depth) were compared pre- and postoperatively and statistically analyzed.

**Results:**

Over the follow-up period, 81.3% of the teeth survived revascularization and regained sensitivity, while 18.7% failed, as they had to be extracted due to serious root resorption. Regarding radiographic outcomes a significant difference could only be found in the decrease of apical foramina (*p* = 0.04). The other parameters showed no significant difference between pre- and postoperative measurements. More than half of the teeth (56.3%) developed root resorptions and 31.3% displayed signs of ankylosis and 92.9% developed discolorations during follow-up. However, 85.7% of the teeth maintained the bone level and outcomes of mobility showed a significant solidification.

**Conclusions:**

Revascularization is a promising approach for the treatment of immature incisors to regain sensitivity and to enhance apical closure and at least to maintain alveolar bone in terms of a socket preservation. Further studies have to be performed to determine ideal conditions (type of trauma, age, width of apical foramen) for a revascularization.

## Background

Dental trauma usually happens when root development is incomplete. Open apices and wide pulps make root canal treatment difficult [1, 2]. Furthermore, due to thin, weak dentinal walls, teeth are more vulnerable to fracture [1].

The traditional treatment of pulp necrosis in immature permanent teeth was a long-term application of calcium hydroxide, which induced an apical hard tissue barrier [2, 3]. However, the aforementioned treatment requires a good compliance, which is related to multiple clinic visits over a long period of time [1]. Therefore, today the conventional treatment for immature teeth with a necrotic pulp is the one-step apexification, in which mineral trioxide aggregate (MTA) is used to create an artificial apical plug [4]. Compared to the calcium hydroxide treatment, the number of appointments is reduced and studies also have shown high clinical success [5]. However, neither of these treatment options allows continued root development [4, 6]. The result is a fragile root structure with a significantly higher risk of cervical root fracture than in mature teeth. According to Cvek, et al. the incidence varies from 28 to 77% depending on the stage of root development [7].

In the early 60s Nygaard-Ostby provided the basis for revascularization, as he described the role of blood clot in apical healing after endodontic therapy [8]. However, Iwaya et al. reported the first case of revascularization in 2001 as an alternative procedure to apexification [9]. From 2001 lots of case reports, case series and review papers were published [10, 11]. They observed not only the healing of periapical lesion, but also an increase in root length and thickness [12–16]. Some studies even described a restoring of pulp vitality [16–19].

In histological studies extracted dog teeth after revascularization treatment were analyzed. The new tissue formation inside the root canal is not pulp parenchymal tissue. Instead of odontoblast and newly formed dentin, tissues resembling cementum, periodontal ligament and bone have been found, which indicates more a healing process than a regeneration [11, 20].

In addition, advanced revascularization techniques have been published in which platelet-rich plasma (PRP) or platelet-rich fibrin (PRF) were applied into the root canal, instead of induction of apical bleeding [21, 22]. In particular PRF should contribute to successful results [23].

Nevertheless, there are still no randomized controlled studies that have shown long-term success [11]. Moreover, case reports generally presented an accumulation of successful outcomes, while there are only a few publications of failed revascularizations [24].

This retrospective study includes cases of revascularization treated by two investigators, following a standardized protocol. Clinical and radiographic data was evaluated and analyzed. We present outcomes of revascularization and thus specify the realistic outcome of revascularization in clinical routine.

## Methods

### Experimental design

The Ethical Review Committee of Charité − University Medicine Berlin formally approved the retrospective analysis of our patient data (EA1/331/16). All parents gave written informed consent to the treatment performed.

Patients with traumatized incisors, lack of vitality 10–14 days after trauma (negative reaction to cold and electric stimulation (VitalityScanner, SybronEndo, Kerr, Brea, USA), an open apex (> 1 mm) and no prior root canal treatment were subject to revascularization therapy. Avulsed teeth’ dry time was less than 30 min and all were stored in a physiologic solution (DentoSafe, Medice, Iserlohn, Germany). Prior to replantation, the anti-resorptive therapy consisted of soaking the tooth into a new storage media enriched with 800 μg doxycyline (50 μm/ml) and 640 μg dexamethasone (40 μm/ml) for 20 min. That time was used to inspect and clean the alveolus with 0.9% physiologic saline solution. Afterwards, the tooth was replanted and splinted with a semi-rigid device with a titanium trauma splint (TTS, Medartis AG, Basel, Schweiz) for 10–14 days.

For the revascularization procedure teeth were isolated using a rubber dam, opened using diamond burs and coronally widened if necessary. Remaining tissue, if present, was necrotic. After passively activated ultrasonic irrigation (PUI) with 10 mL of 1% sodium hypochlorite and 2 mL of 17% ethylenediaminetetraacetic acid (EDTA, Coltène, Altstaetten, Switzerland) an intermediate dressing of demeclocycline (tetracycline) and triamcinolone (cortisone) (Ledermix®, Greifswald, Germany) was applied. Cavities were closed with Cavit® (3 M Espe, MN, USA) and a glass ionomer cementum. After 7 to 10 days the procedure of revascularization was performed if teeth did not display any symptoms such as pain or swelling. The teeth were anesthetized using 4% articaine hydrochloride without vasoconstrictor (Ultracain® D) and isolated using rubber dam. Revascularization was performed using the dental microscope for all steps. Teeth were rinsed with 5 mL EDTA using PUI, irrigation was performed with an endo irrigation needle with the diameter of 0.3 (Transcodent, Kiel, Germany) and subsequently root canals were dried using paper points. With the help of a sterile ISO 10 C-Pilot file (vdw GmbH, Munich, Germany) bleeding was induced by slight over-instrumentation. After approximately 5 min, a manually individualized sterile collagen sponge (collacone, Botiss Dental, Berlin, Germany) was applied 3–4 mm below the cement-enamel junction to create an abutment for the insertion of mineral trioxide aggregate (ProRoot MTA, Dentsply Sirona, York, USA). A coronal MTA plug of 3 mm was applied below the cement-enamel junction at first third of the root using the MAP system needles (Dentsply Sirona). An X-ray image was performed in order to check the coronal plug and the cavity was adhesively closed using primer and adhesive (OptiBond FL, Kerr, Brea, USA) and a dual cure composite (Luxacore, DMG, Hamburg, Germany). Recalls were performed after approximately 6 months and then on a yearly basis.

### Radiographic analysis

**Fig. 1:**
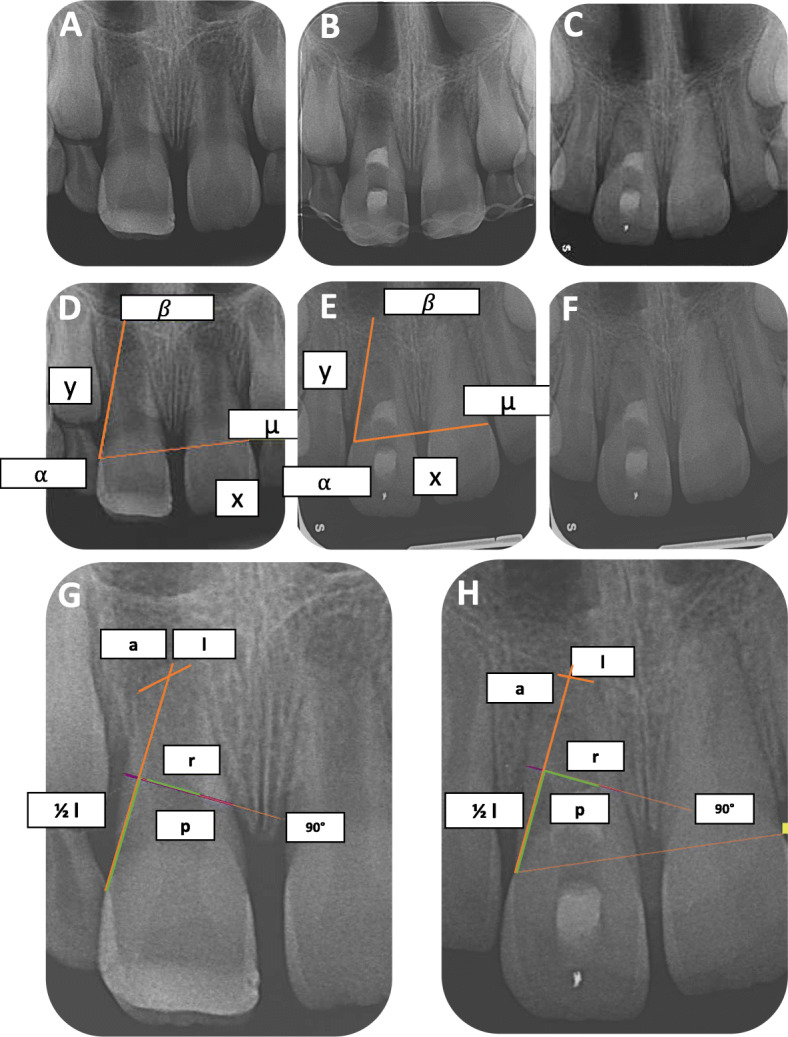
*A representative case:***A** Preoperative radiograph of a necrotic, immature teeth with an open apex of an 8-year-old girl. **B** Radiograph directly after the treatment to check the coronal MTA plug. **C** Postoperative radiograph after 10-month follow-up period showing disappearance of periapical radiolucency, apical closure and distal sign of resorption at the coronal third of the root. Selected landmarks (α, β, μ) on the preoperative (**D**) and postoperative radiograph (**E**) to adapt the postoperative radiograph and set the scale (x,y). **F** The corrected postoperative radiograph after using TurboReg plug-in application of ImageJ. **G** The measurement of the preoperative radiograph. **H** The measurement of the postoperative radiograph. The length (l) increased from 12.71 mm to 13.51 mm, the root thickness (r) increased from 6.12 mm to 6.27 mm and the pulp space (p) increased from 3.29 mm to 3.38 mm. The size of the apical diameter (a) decreased from 3.24 mm to 2.09 mm

The preoperative and final recall radiographs were generated by a direct or indirect digital X-ray system. All images were measured independently by two different investigators who did not perform the treatment. For measurement and recording we used SIDEXIS XG (Dentsplay Sirona). Furthermore, we utilized the software ImageJ with the TurboReg plug-in to minimize distortions, caused by slightly different angulation of the X-ray central beam between the preoperative and postoperative radiographs (Fig. 1). The retrospective study by Bose et al. served as a base for radiographic analysis in this study [25]. In summary, one image was selected as the source and the other as the target, which was adapted to the source image. In the present study the final recall X-ray images were adjusted to the preoperative radiographs. In addition, on both three identical landmarks were selected, which had to fulfill three criteria (Fig. 1: α, β, μ). They had to be constant over the time, clearly defined and easily visible. One case could not be included, as no constant landmarks could be determined. After selection, the TurboReg “automatic mode,” Image J corrected the target image [13, 26] (Fig. 1). Eventually, the corrected image was imported to SIDEXIS XG again. Its scale was calibrated by choosing two reference points on the preoperative image and by using the “set scale” option in SIDEXIS XG. After standardization of the radiographs the teeth were measured (Fig. 1). At first, a straight line from the cement-enamel junction (CEJ) to the radiographic apex of the teeth represented the root length (Fig. 1: l) [6, 13, 15]. Thereafter, on the half of the root length a right-angle straight line was dragged. Along this line the root thickness and pulp space were measured (Fig. 1: r, p). The difference between the pulp space and the root thickness determined the dentin wall thickness. The apical diameter was measured by dragging a straight line across the radiographic apical foramen (Fig. 1: a). The preoperative and postoperative radiographs were also evaluated as to whether they show a presence or absence of periapical radiolucency, sign of root resorption, marginal bone resorption or ankylosis.

### Statistical analysis

Data were imported in SPSS 23.0 (IBM, Armonk, USA) in order to determine the significance.

Caused by the low number of cases and non-normal distribution, non-parametric tests were used. For comparison of the metric dates, the Wilcoxon test was utilized and the significance of the nominal scaled dates were tested with the chi-square test (cross-table). Radiographic measurements, which were performed by two different investigators, were compared regarding differences by using the Mann−Whitney U test. *P* values < 0.05 were considered significant.

## Results

We included 12 patients and 16 teeth (Table 1). The study includes four patients, who got two teeth treated by revascularization. However, each tooth was evaluated as an individual case. The average age of the patients was 10 years and the gender was balanced (47% female, 53% male). The etiology of the necrotic pulp was either avulsion or serious luxation / intrusion. One patient had to be excluded because of non-compliance with recall. Of 16 recalled teeth (18.8%) 3 had to be extracted after the listed follow-up period, because the X-ray image showed signs of serious root resorption. Therefore, 81.3% of the teeth survived revascularization and 18.8% failed.

**Table 1:** Patient’s demographic data

Patient no.	Age range (y)	Tooth no.	Trauma	Follow-up (month)	Reasons for failure/ exclusion
1	8–9	9	Avulsion	28	
2	10–11	8	Avulsion	14	
2	10–11	7	Luxation	14	
3	10–11	8	Luxation	23	
3	10–11	9	Avulsion	38	
4	6–7	8	Avulsion	54	
5	8–9	8	Luxation	27	
5	8–9	7	Avulsion	Failed after 27	Extracted due to serious rout resorption
6	8–9	9	Avulsion	32	
7	10–11	8	Avulsion	33	
8	8–9	8	Avulsion	10	
9	10–11	8	Avulsion	15	
10	6–7	8	Intrusion	11	
11	10–11	7	Avulsion	12	
12	10–11	9	Avulsion	Excluded	Missed the recall
13	10–11	8	Avulsion	Failed after 4	Extracted due to serious rout resorption
13	10–11	8	Avulsion	Failed after 10	Extracted due to serious rout resorption

### Radiographic outcomes

**Fig. 2:**
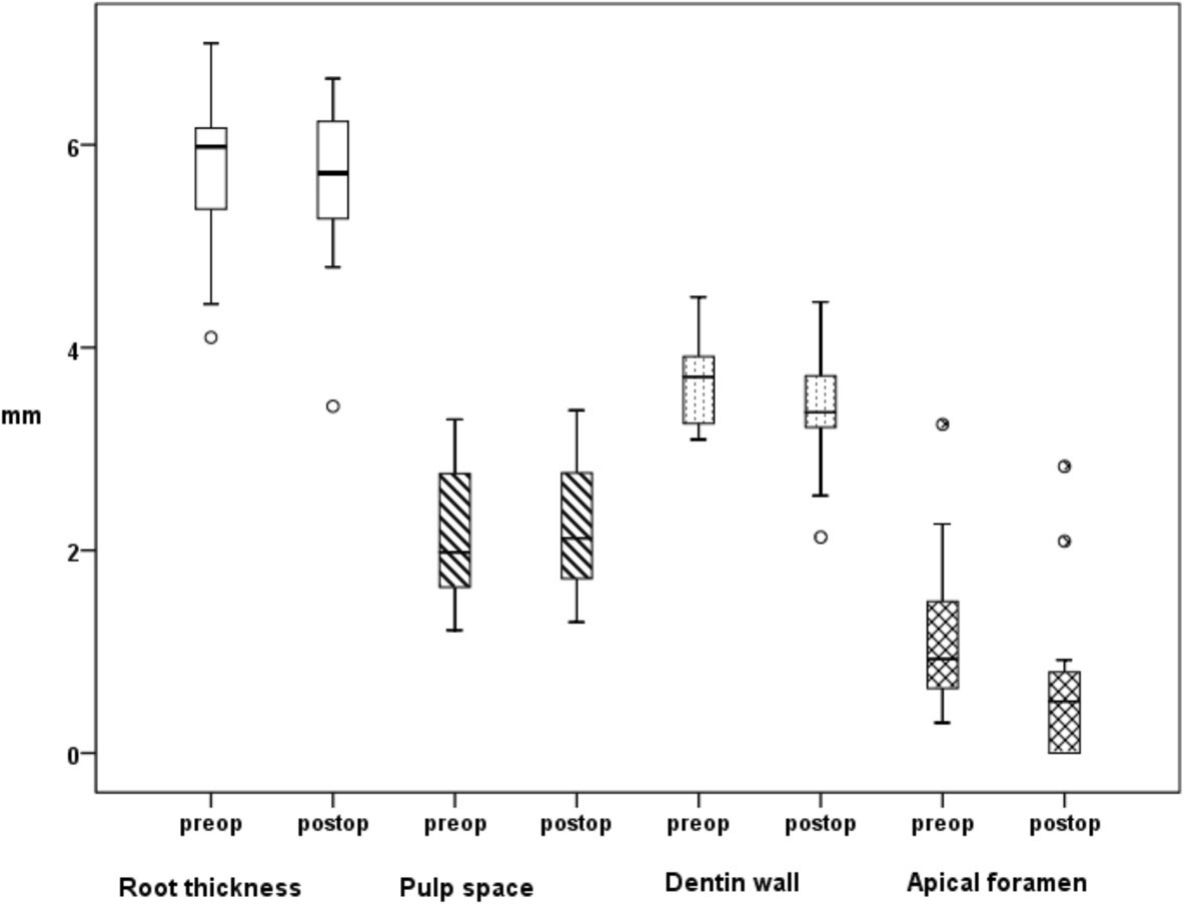
*Boxplot showing teeth development.* Changes in root thickness, pulp space and dentin wall widths were not significant, a significant difference could be found in the size of the apical foramina

**Fig. 3:**
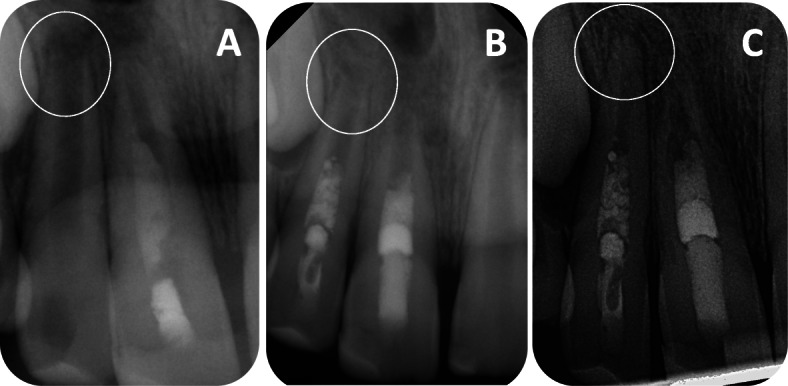
*A representative case:***a** Preoperative radiograph of a necrotic, immature tooth with an open apex of a 10-year-old boy. **b** Radiograph directly after the treatment to check the coronal MTA plug. **c** Postoperative radiograph after 14-month follow-up period showing disappearance of periapical radiolucency and apical closure. The development of the apical diameter is marked with a circle

The evaluation of the accordance between the independent investigators revealed no significant differences. The radiographic difference of the root thickness, pulp space, dentin wall widths and apical foramen between the pre- and postoperative X-ray images in mm are presented in Fig. 2. A significant difference was only found in the decrease of apical foramina (Fig. 3). After about 7 months, a decrease in the size of the apical foramina was radiologically visible. The other parameters showed no significant difference between pre- and postoperative measurements. Regardless of the different teeth size, the development of the teeth is displayed as a value in percent in Table 2. The pulp space showed a tendency of a 9.97% increase and dentin thickness a tendency of a 6.91% decrease. But *p values* of 0.27 and 0.11 indicated no significance. Moreover, the teeth increased in root length on an average by 0.96% during the follow-up period, without showing significance. Only the 36.94% decrease of the apical foramen diameter compared to the initial situation is significant (*p* = 0.04).

**Table 2:** Average teeth development in percent

	Root length	Pulp space	Dentin wall	Apical foramen
**Mean**	0.96%	9.97%	−6.91%	−36.94%
**Standard deviation**	9.61%	26.78%	12.36%	57.42%
***P*value**	.87	.27	.11	**.04**

The Wilcoxon test was used to test the significance

### Clinical outcomes

**Fig. 4:**
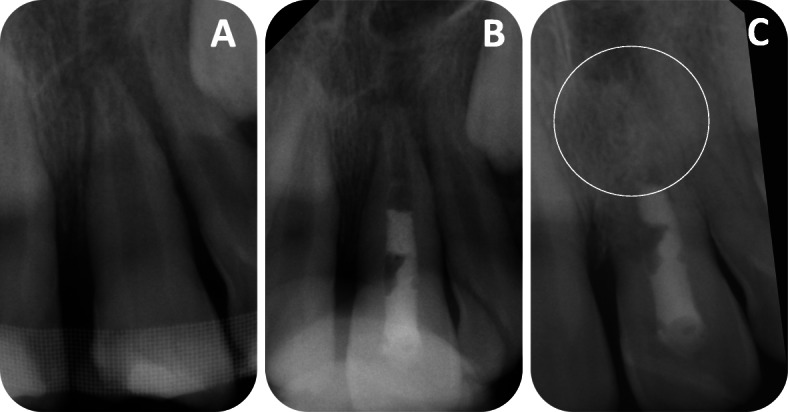
*A representative case:***a** Preoperative radiograph of a necrotic, immature tooth with an open apex of a 9.5-year-old boy. **b** Radiograph directly after the treatment to check the coronal MTA plug. **c** Postoperative radiograph after 33-month follow-up period showing replacement resorption/ankylosis, which is marked with a circle

Several clinical pre- and postoperative parameters were compared to analyze the recovery of the treated teeth (Table 3, 4 and 5)*.* Statistical analysis demonstrated in 75% of cases, which had a periapical radiolucency preoperatively, that the radiolucency was absent directly after treatment. Nevertheless, 16.7% of the teeth without a periapical radiolucency preoperatively developed a periapical radiolucency. Moreover, no teeth showed tenderness to percussion after the last recall. Most of the treated teeth (81.3%) regained sensitivity during the follow-up period. Nevertheless, more than half of the teeth developed a root resorption after the treatment (56.3%) and 31.3% of the treated teeth displayed signs of ankylosis postoperatively (Fig. 4). Every tooth with a loss of marginal bone before treatment showed a physiological level of alveolar bone after the follow-up period. However, if the teeth had no sign of marginal bone loss preoperatively, 14.3% of them lost alveolar bone during the follow-up period and 85.7% of them maintained the level of marginal bone.

**Table 3:** Clinical outcome measures

	Preoperative remarkable		Preoperative unremarkable		*P* value
	Improved	Still remarkable	Deteriorated	Still unremarkable	
Periapical radiolucency	75.00%	25.0%	16.7%	83.3%	0.712
Alveolar bone loss	100.0%	0.0%	14.3%	85.7%	0.568
Root resorption	0%	0%	56.3%	43.8%	
Ankylosis	0%	0%	31.3%	68.8%	
Supra−/infraposition*	0.0%	100.0%	23.1%	76.9%	**0.0133**
Sensitivity	81.3%	18.8%	0%	0%	
Percussion	100.0%	0%	0%	100.0%	
Discoloration	0.0%	100.0%	92.9%	7.1%	0.7827

The chi-square test was used to test the significance

**Table 4:** Mobility

		Median	57% Percentile	***P*** value
Mobility^a^	preoperative	2	2	**.013**
	postoperative	0	1	

The Wilcoxon test was used to test the significance

^a^The mobility was evaluated on a scale from zero (fixed teeth) to three (high mobility)

**Table 5:** Probing depth

		Mean	Standard deviation	***P*** value
Probing depth^a^	Preoperative	3.625	.8062	.259
	Postoperative	3.281	1.2776	

The Wilcoxon test was used to test the significance

^a^The probing depth was measured at the deepest point

Regarding the tooth position, a significant development was found. If the tooth was in a supra- or infra-position before treatment, it did not change position in 100% of the cases. But 23.1% of the teeth, without a sign of infra- or supra-position preoperatively, were not in their normal position after treatment compared to adjacent teeth.

In addition, 92.9% of the teeth, which had a normal color before, developed discolorations during the follow-up period. Outcomes of mobility showed a significant solidification (Table 4) and the probing depths showed a tendency to decrease (Table 5)*.*

## Discussion

The overarching aim of revascularization of a traumatized immature tooth is to preserve teeth as long as possible. The loss of permanent teeth at young ages could lead to breakdown and growth arrest of the alveolar bone, which impedes a later esthetical and functional reconstruction [11]. Furthermore, for esthetic purposes it is not recommended to implant until dental and skeletal growth have been completed [27]. Based on our underlying study, most teeth (81.3%) survived after the average follow-up period of 22 month. The teeth mobility decreased significantly, the probing depths were reduced and none of the teeth showed tenderness to percussion after the follow-up period. Although the decrease of the probing depth was not significant, the teeth were functional again.

In case of a preoperative apical radiolucency caused by trauma, 75% of our underlying cases showed no more periapical radiolucency on the radiograph which has been taken on the day of revascularization procedure, indicating a reattachment of the tooth before revascularization.

Almost all teeth (92.9%) became darker during the follow-up period due to the use of intracanal medicament Ledermix, which is known to discolor teeth, especially immature teeth [28, 29]. Moreover, in some studies, MTA has caused for discoloration, which we used as a coronal seal [30, 31]. In particular, the contamination of MTA and blood increased teeth discoloration [31, 32]. In the underlying study one tooth did not become darker as MTA and Ledermix were successfully placed only below the gingiva margin, thereby averting the esthetic disadvantage of discoloration [28].

At the end of the follow-up period, 81.3% of the cases responded to the pulp sensibility test (cold or electric) again, which could be an indicator of the regeneration of nerve tissue. However, the teeth in our study were not examined histologically. Previous histological studies of human and animal teeth did not find a typical pulp tissue in the root canal, more a tissue similar to periodontal ligament and a cementum-like or bone-like hard tissue [20, 33]. It is assumed that this endodontic procedure led more to a healing or repairing as to a regeneration of pulp tissue [11]. Therefore, our underlying study is based on the assumption that new tissue includes nerve fibers as the most vital tissue does [13, 34]. However, the usual transmission of impulses relying on hydrodynamic mechanisms is improbable.

In case of avulsion, a pulp healing without revascularization treatment is possible [35], while severe root resorptions can proceed rapidly [36]. We therefore decided to initiate treatment, if teeth showed lack of vitality for 10–15 days.

Although we used Ledermix as an intracanal medicament, which has been shown to inhibit extern root resorption [37], 56.3% (9 teeth) of the cases in the underlying study showed resorption after the follow-up period. Resorption is classified into replacement resorption/ankylosis, surface resorption and inflammatory resorption [38, 39]. Five cases out of 9 that showed resorption had been ankylosed, which was visible on the X-ray image as a disappearance of the periodontal space (31.3% of all treated teeth) (Fig. 4) [38]. According to the literature, ankylosis is a consequence of periodontal ligament and pre-cement loss [40–42]. If the periodontal ligament is damaged and therefore the root surface is in direct contact with the alveolar bone, the tooth will be resorbed by osteoclast and replaced by bone tissue [40, 42]. Animal experimental studies have shown that 2 × 2 mm of root defects can heal [40]. As the cavity is larger, the process can finally lead to tooth loss [43], which is applicable in two failing cases in the underlying study. However, Panzarini et al. described ankylosis as the best result as there are no periodontal ligament remnants [43]. The unavoidable effect of tooth loss can be delayed and therefore the atrophy of the alveolar ridge is prevented, which simplifies later implantation [27]. Moreover, ankylosis explains the changes in tooth position compared to the adjacent teeth in 23.1% of our cases. If ankylosis occurs during the growth process of a patient, it will result in no further tooth eruption caused by the loss of periodontal membrane [44, 45]. Therefore, in the underlying study, ankylosis became visible through infraposition.

Surface resorption, also called healing-related resorption, is one of the favorable types [46] as it self-limited and not progressive, provided that the cavity is confined to the cementum or the pulp is not necrotic [47]. In three of the resorption cases, the resorption was visible on the X-ray image. As the resorption was controllable and was not progressive during the follow-up period, this indicates a surface resorption (Fig. 1). Probably trauma, especially avulsion will always lead to minimal injury to the periodontal ligament and thus at least to surface resorption [36]. However, due to the small size, surface resorption is not always visible on the X-ray image [36]. We can therefore assume, that in 43.7% of the cases, in which no absorption was detected, the surface absorption was only not visible on the X-ray image.

Inflammatory resorption occurs if pulp is infected and toxic elements diffuse from the pulp canal to the resorption cavity of the periodontal ligament damage and contamination [39]. The extraction of the infected tooth might be avoided and the absorption can be arrested by early endodontic treatment [38, 48]. One of the failed cases, which needed to be extracted due to serious root resorption, was inflammatory. In this regard the possibility to retread the tooth endodontically was tardy due to irregular recalls of the patient.

Following the aforementioned facts, resorption is more likely to be caused by the trauma than by the revascularization treatment. Avulsion in particular has the lowest healing rate and a high prevalence of root resorption [49]. Replacement root resorption has the highest incidence after avulsion followed by inflammatory root resorption and surface root resorption [50].

Our underlying study focused on the standardized radiographic analysis to get the most accurate possible data of the hard tissue development after revascularization therapy. In many case reports and case series of revascularization, it is described that a continued root development with new hard tissue formation occurred [9, 12, 13, 16, 51]. The realistic aim of revascularization is limited in our underlying study for the following reasons. The average increase in 0.15% of root length was not significant and therefore cannot be interpreted as a continuation of root development. The slightly average increase in 0.15% of pulp space and average decrease in 0.26% of dentin wall indicated a hard tissue loss. On the one hand the loss could have been caused by the cautious instrumentation of the root canal to eliminate the necrotic tissue and on the other hand, in the cases of inflammatory or replacement resorption, tissue remodeling could have led to a degradation of dentin wall.

The analysis reveals a significant change in the apical diameter (Fig. 3). The postoperative X-ray images showed an average closure of 0.47%. Therefore, some hard tissue formed at the apical foramen. In conclusion, with regard to our results only the significant apical closure can be anticipated after revascularization treatment. However, studies have shown that this can also be reached by a long-term application of calcium hydroxide or artificially by a one-step apexification [3, 4]. This result is confirmed by the cohort study of Alobaid et al. According to the cohort study there is no significant superiority of revascularization compared to other apexification therapies [26]. Moreover, our underlying study revealed no significant progress in root development. Thus, no advantage of fracture resistance can be expected.

Nevertheless, for a final evaluation further studies are necessary and a standardized recall with regular intervals would be desirable to analyze the root development dependent on time. With regards to the interpretation in root development, 2 years of follow-up are sufficient.

Moreover, not all pre- and postoperative radiographs were taken with the same angulation. Even though we used TurboReg plug-in application of Image J to minimize the deviation, this step might be most critical. We paid particular attention to setting the landmarks, which were used to match the pre- and postoperative X-ray images by Image J. If there were no points found that were constant over time, clearly defined and easily visible, the case was excluded from the investigation.

Hitherto, many case reports and case series viewed revascularization very positively, as the studies observed an increase in root length and dentin wall thickness [9, 12–14, 16, 19]. The discrepancies in our study can be explained based on the following issues. Many studies have a lower number of cases [12, 16–19], a shorter period of follow-up [13, 18, 19] or no standardized and/or blinded radiographic evaluation [14, 24]. Moreover, most analyses include teeth with diverse etiologies of necrotic pulp [14, 15, 24]. For instance, caries can lead to pulp infection and may result in an interruption of root development, which represents an indication for revascularization therapy as well [11]. In the aforementioned cases, great damage to periodontal ligament, which was shown to be a reason for root resorption, is very unlikely, which explains our high rate of resorption compared to other case reports. Moreover, re-implanted teeth have a higher risk of Hertwig’s epithelial root sheath (HERS) or apical papilla injury [13, 52], which are described as the most important elements to determine the continuing of root development after a severe trauma [14, 52, 53]. Thus, traumatized teeth, especially avulsed teeth, are less likely to complete root development after revascularization than teeth with necrotic pulp caused by caries [13].

Published studies have not demonstrated so far which steps of the protocols are worthy of improvement [11]. Therefore, more studies should be conducted to optimize revascularization protocol, which has predictable and ideal outcomes. Promising approaches are shown in the field of tissue engineering, using stem cells, customized scaffolds and growth factors to manage the tissue responses.

## Conclusion

The underline study shows revascularization as an appropriate therapeutic approach for traumatized immature incisors to regain sensitivity and to enhance apical closure and at least to maintain alveolar bone in terms of a socket preservation. However, a complete root development in length and thickness cannot be expected and the prognosis for the teeth is limited by the risks of trauma.

## Data Availability

The datasets used are available from the corresponding author on reasonable request.

## Abbreviations

MTA: Mineral trioxide aggregate
PRP: Platelet-rich plasma
PRF: Platelet-rich fibrin
TTS: Titanium trauma splint
PUI: Passively activated ultrasonic irrigation
EDTA: Ethylenediaminetetraacetic acid
MAP: Micro-Apical Placement
CEJ: Cement-enamel junction
HERS: Hertwig’s epithelial root sheath
